# Low-moderate arsenic exposure and respiratory in American Indian communities in the Strong Heart Study

**DOI:** 10.1186/s12940-019-0539-6

**Published:** 2019-11-28

**Authors:** Martha Powers, Tiffany R. Sanchez, Maria Grau-Perez, Fawn Yeh, Kevin A. Francesconi, Walter Goessler, Christine M. George, Christopher Heaney, Lyle G. Best, Jason G. Umans, Robert H. Brown, Ana Navas-Acien

**Affiliations:** 1 Department of Environmental Health and Engineering, Johns Hopkins Bloomberg School of Public Health, 615 N. Wolfe St, Baltimore, MD USA; 2 Department of Environmental Health Sciences, Columbia University Mailman School of Public Health, New York, NY USA; 3 Center for American Indian Health Research, University of Oklahoma Health Sciences Center, College of Public Health, Oklahoma City, OK USA; 4 Institute of Chemistry - Analytical Chemistry, Graz, Austria; 5 Department of International Health, Johns Hopkins Bloomberg School of Public Health, Baltimore, MD USA; 6 Department of Epidemiology, Johns Hopkins Bloomberg School of Public Health, Baltimore, MD USA; 7 Missouri Breaks Industries Research, Inc., Eagle Butte, SD USA; 8 MedStar Health Research Institute, Hyattsville, MD, USA, Georgetown-Howard Universities Center for Clinical and Translational Science, Washington, DC USA; 9 Department of Anesthesiology and Critical Care Medicine, Johns Hopkins School of Medicine, Baltimore, MD USA

## Abstract

**Background:**

Arsenic exposure through drinking water is an established lung carcinogen. Evidence on non-malignant lung outcomes is less conclusive and suggests arsenic is associated with lower lung function. Studies examining low-moderate arsenic (< 50 μg/L), the level relevant for most populations, are limited. We evaluated the association of arsenic exposure with respiratory health in American Indians from the Northern Plains, the Southern Plains and the Southwest United States, communities with environmental exposure to inorganic arsenic through drinking water.

**Methods:**

The Strong Heart Study is a prospective study of American Indian adults. This analysis used urinary arsenic measurements at baseline (1989–1991) and spirometry at Visit 2 (1993–1995) from 2132 participants to evaluate associations of arsenic exposure with airflow obstruction, restrictive pattern, self-reported respiratory disease, and symptoms.

**Results:**

Airflow obstruction was present in 21.5% and restrictive pattern was present in 14.4%. The odds ratio (95% confidence interval) for obstruction and restrictive patterns, based on the fixed ratio definition, comparing the 75th to 25th percentile of arsenic, was 1.17 (0.99, 1.38) and 1.27 (1.01, 1.60), respectively, after adjustments, and 1.28 (1.02, 1.60) and 1.33 (0.90, 1.50), respectively, based on the lower limit of normal definition. Arsenic was associated with lower percent predicted FEV1 and FVC, self-reported emphysema and stopping for breath.

**Conclusion:**

Low-moderate arsenic exposure was positively associated with restrictive pattern, airflow obstruction, lower lung function, self-reported emphysema and stopping for breath, independent of smoking and other lung disease risk factors. Findings suggest that low-moderate arsenic exposure may contribute to restrictive lung disease.

## Introduction

Arsenic exposure via drinking water is a well-established lung carcinogen [1–3]. More recently, water arsenic > 100 μg/L has been associated with non-malignant respiratory effects, including respiratory symptoms and worse lung function tests. A recent meta-analysis identified an association between arsenic exposure and reduced forced vital capacity (FVC) and forced expiratory volume in 1 s (FEV1) with a preserved ratio (in subset of 3 studies reporting FEV1/FVC), indicating a possible association with restrictive lung disease [4]. The studies in the meta-analysis included a wide range of exposure levels, with arsenic often 10 times higher than the World Health Organization guideline/United States Environmental Protection Agency standard of 10 μg/L in drinking water. More evidence is needed at low-moderate levels of arsenic exposure (< 50 μg/L), and levels common in the US and other countries (< 10 μg/L). A recent systematic review showed strong evidence of an association between high levels of arsenic exposure with respiratory symptoms, non-malignant respiratory illness, and reduced lung function [5]. One study from the US found no association between low-moderate arsenic exposure and self-reported diagnosis or symptoms of obstructive lung disease but lacked spirometry data [6]. We examined the association of low-moderate arsenic exposure with respiratory health in American Indians from the Northern Plains, the Southern Plains and the Southwest United States, communities with environmental exposure to inorganic arsenic through drinking water.

## Methods

### Study population

The Strong Heart Study (SHS) is an ongoing population-based, prospective study of cardiovascular disease and its risk factors in American Indian adults. The SHS recruited 4549 residents of Tribal Nations from study sites located in Arizona (AZ), Oklahoma (OK), and North Dakota and South Dakota (ND/SD) in the US. Study enrollment rates were 71.8% in AZ, 61.5% in OK, and 55.3% in ND/SD [7]. All men and women aged 45 to 74 years at the baseline visit in 1989–1991 were invited to participate, with subsequent clinical visits [8]. In 2016, one community in Arizona withdrew their consent, reducing the cohort to 3516 participants. To account for the unintended withdrawal of a Tribal Nation, all analyses were weighted using inverse probability weighting. As study site proportion is known from the original cohort, the withdrawal of the Tribal Nation was adjusted for by weighting the remaining participants, with approximately 1/3 of weight for each center (33.0% AZ, 33.6% OK, 33.4% ND/SD); the use of the statistical weight is to reduce bias introduced by drop-out [9].

This study uses urinary arsenic data from the baseline examination and spirometry from Visit 2 (1993–1995), both available in 2271 participants. We excluded 94 participants missing baseline data on smoking status and cigarette pack-years, 11 missing diabetes status, education, or body mass index (BMI), and 34 missing tuberculosis data, leaving 2132 participants.

### Data collection

Visits included biospecimen collection, physical exam, and an interviewer-administered standardized questionnaire. Visits were performed by trained and certified examiners. Details have been described previously [8].

### Urine arsenic

Morning spot urine samples were collected at baseline [8]. For arsenic analyses, urine concentrations of inorganic arsenic (iAs), methylarsonate (MMA), and dimethylarsinate (DMA) were measured using high performance liquid chromatography/inductively coupled plasma-mass spectrometry. The metabolism of inorganic arsenic in the human body results in MMA and DMA which are excreted in urine together with unchanged inorganic arsenic. Quality control and assurance methods and laboratory procedures for urine analysis have been described [10]. We used the sum of inorganic and methylated arsenic species (iAs + MMA + DMA) as the biomarker of exposure to inorganic arsenic in drinking water and food. Arsenobetaine levels are low in the population (median (10th, 90th percentiles): 0.5 μg/g (< 0.6–6.10) creatinine], confirming that seafood intake is rare [11]. Urine arsenic concentrations (μg/L) were divided by urine creatinine concentrations (g/L) to account for urine dilution in spot urine samples and expressed as concentrations of total urine arsenic and its species in μg/g creatinine.

### Spirometry for identification of airflow obstruction and restrictive pattern

Spirometry was performed by trained and certified nurses and technicians [12]. Pre-bronchodilator testing was conducted while sitting, except for participants with BMI > 27 kg/m^2^ who stood. Maneuvers were considered acceptable to then-current American Thoracic Society recommendations [12, 13].

Spirometry metrics FEV1, FVC, and FEV1/FVC were used in analyses. Reference values for SHS participants were derived previously [12] yielding FVC %predicted and FEV1%predicted. The prevalence of airflow obstruction was defined by a fixed ratio of FEV1/FVC < 0.70 using crude values [14]. A low FVC (< 80%predicted) together with a preserved ratio (FEV1/FVC ≥ 0.70) was defined as restrictive pattern [15]. Healthy individuals (controls) were those with no-obstruction and no-restriction (FEV1/FVC > 0.70 and FVC > 80%predicted). We conducted secondary analyses with the lower limit of normal (LLN = 5th percentile of the frequency distribution of reference values; obstruction: FEV1/FVC < LLN; restriction: FEV1/FVC > LLN and FVC < LLN; healthy: FEV1/FVC > LLN and FVC > LLN).

### Symptoms and lung disease

At Visit 2, participants were asked to report respiratory symptoms including cough (“Do you usually have a cough?”, frequent cough (“Do you usually cough as much as 4-6 times/day, 4 or more days/week?”), cough with phlegm (“Do you usually bring up phlegm when you cough?”), shortness of breath (“Are you troubled by shortness of breath when hurrying on the level or walking up a slight hill?”), and stopping for breath while walking (“Do you ever have to stop for breath while walking about 100 yards or a few minutes on the level?”). Participants self-reported if a medical person ever told them they had emphysema, asthma, or chronic bronchitis diagnoses, which was recorded at Visit 2.

### Other variables

At the baseline visit, sociodemographic (age, sex, education, and study site) and life-style (smoking status and smoking pack-years) variables were ascertained through a standardized questionnaire by trained and certified interviewers [8]. Smoking status was categorized as never, former, or current. Former: smoked ≥100 cigarettes but no longer smoking; Never: smoked < 100 cigarettes in lifetime; and Current: smoking at then-present day. Height and weight measurements for BMI calculation (weight in kilograms divided by height in meters squared) were conducted during the physical exam. Chronic kidney disease was defined as estimated glomerular filtration rate (eGFR) < 60 ml/min/1.73m^2^ based on serum creatinine using the Modification of Diet in Renal Disease equation [16]. Diabetes was defined as a fasting glucose level of ≥126 mg/dL, a 2-h post-load plasma glucose level of ≥200 mg/dL, an HbA1c level of ≥6.5%, or use of an oral hypoglycemic agent or insulin [17].

At Visit 2, a medical record review for a history of active and treated tuberculosis (class III tuberculosis) was performed. Case definition for class III tuberculosis involved having a positive culture for *Mycobacterium tuberculosis* from a body fluid or tissue or having a clinical picture suggestive of tuberculosis that responded to treatment with antitubercular medications. If the individual had active tuberculosis listed on a discharge diagnosis or on a problem list, they were considered to have a history of tuberculosis.

### Statistical analysis

We conducted descriptive statistics to evaluate differences in participant demographic and lifestyle variables by obstruction and restrictive pattern and by urinary arsenic tertile. We used logistic regression to estimate the odds ratio [OR] for presence of obstruction/restrictive pattern, respiratory symptoms and disease by urinary arsenic concentrations, and linear regression to assess the mean difference of spirometric measurements. We modelled arsenic exposure using three approaches: a categorical variable, comparing tertiles of arsenic exposure; a continuous variable to compare an interquartile (IQR) increase of log urinary arsenic; and a continuous variable with splines with knots at the 10th, 50th, and 90th percentiles (3.8, 10.2, and 25.8 μg/g creatinine, respectively) to allow for a flexible dose-response relationship. *P* values for trend were obtained from modelling log-arsenic as continuous. Models were progressively adjusted (see footnotes of Tables 3, 4, 6).

Effect modification of the association was evaluated depending on confounding variables by including interaction terms for log-transformed urinary arsenic concentrations with indicator variables for sex, age, smoking status, BMI, and diabetes. *P* values for interactions were obtained using Wald test for multiple coefficients. To evaluate arsenic metabolism, we examined the association between the relative proportions of arsenic species in urine per 5% change and presence of obstruction/restrictive pattern.

## Results

Obstruction was present in 21.5% (458/2132) and restrictive pattern present in 14.4% (307/2132). Obstruction and restrictive pattern demographics are described in Table 1. Obstruction was present in 31.0% vs. 20.7% of participants in the highest vs. lowest arsenic exposure tertiles (*p* = 0.02); restrictive pattern was present in 23.8% vs. 17.2% of participants in corresponding tertiles (*p* < 0.001) (Table 2).

**Table 1:** Baseline (1998–1991) Participant Characteristics by Airflow Obstruction and Restrictive Pattern at Visit 2 (1993–1995) (*N* = 2132)

	Airflow obstruction	Restrictive Pattern	Healthy
	FEV1/FVC < 0.70 (*n* = 458)	FEV1/FVC > 0.70 FVC < 80% predicted (*n* = 307)	FEV1/FVC > 0.70 FVC > 80% predicted (*n* = 1367)
Age, years	59.2 (0.3)	56.1 (0.5)	54.5 (0.2)
Female	226 (49.3%)	206 (67.1%)	860 (62.9%)
Education			
No high school (HS)	122 (26.6%)	68 (21.1%)	187 (13.7%)
Some HS	111 (24.2%)	69 (22.4%)	313 (22.9%)
Completed HS or higher	225 (49.2%)	170 (55.4%)	867 (63.4%)
Smoking status			
Never	113 (24.7%)	109 (35.5%)	451 (33.0%)
Former	134 (29.3%)	91 (29.6%)	443 (32.4%)
Current	211 (46.1%)	107 (34.9%)	473 (34.6%)
Smoking pack years	18.2 (0.9)	9.2 (0.7)	8.2 (0.3)
BMI, kg/m^2^	29.0 (0.2)	33.0 (0.4)	31.4 (0.2)
Diabetes	168 (36.7%)	185 (60.3%)	539 (39.4%)
Urine ∑As, μg/g creatinine	11.1 (6.2–16.1)	12.0 (6.2–20.2)	9.5 (5.6–15.7)
iAs, %	8.5 (5.9–11.7)	7.6 (5.6–10.7)	7.7 (5.5–11.0)
MMA, %	14.7 (11.3–18.5)	12.8 (10.5–16.2)	13.8 (10.8–17.2)
FEV1, %predicted	77.7 (0.8)	73.6 (0.6)	100.2 (0.3)
FVC, %predicted	93.9 (0.8)	69.4 (0.5)	98.6 (0.3)
FEV1/FVC, %	62.2 (0.3)	81.4 (0.4)	78.2 (0.1)
Inhaled steroids	8 (1.7%)	8 (2.6%)	36 (2.6%)
Self-reported chronic bronchitis	59 (13.0%)	49 (16.1%)	112 (8.3%)
Self-reported emphysema	34 (7.5%)	19 (6.3%)	25 (1.8%)
Self-reported asthma	59 (13.1%)	40 (13.1%)	87 (6.4%)
Medical record tuberculosis	83 (18.1%)	54 (17.6%)	155 (11.3%)
Self-reported cough	140 (30.6%)	84 (27.5%)	252 (18.5%)
Cough 4–6x/week	92 (64.8%)	54 (63.5%)	158 (61.5%)
Phlegm	89 (62.2%)	51 (60.0%)	161 (59.9%)
Shortness of breath	221 (48.6%)	169 (57.1%)	585 (43.1%)
Stopping for breath	97 (41.8%)	72 (42.4%)	186 (31.3%)

All analyses are weighted. Data are mean (SE), *n* (% of column), or median (interquartile range)

∑As = inorganic arsenic plus methylated species

**Table 2:** Participant Characteristics by Baseline (1998–1991) Urinary Arsenic Concentration (*N* = 2132)

	Inorganic Plus Methylated Arsenic Species μg/g creatinine			*P*-value^b^
	Tertile 1	Tertile 2	Tertile 3	
	≤7.0^a^	7.1–13.9^a^	≥14.0^a^	
Age, years	55.6	55.6	56.0	0.37
Female	494 (56.0%)	443 (61.1%)	355 (67.6%)	< 0.001
Education				< 0.001
No high school (HS)	80 (9.1%)	149 (20.6%)	148 (28.2%)	
Some HS	202 (22.9%)	160 (22.1%)	131 (25.0%)	
Completed HS or higher	600 (68.0%)	416 (57.4%)	246 (46.9%)	
BMI, kg/m^2^	30.8 (0.1)	31.3 (0.2)	31.6 (0.3)	0.01
Diabetes	317 (35.9%)	296 (40.8%)	279 (53.1%)	< 0.001
Smoking status				0.06
Never	292 (33.9%)	214 (29.5%)	167 (31.8%)	
Former	288 (32.6%)	235 (32.4%)	145 (27.6%)	
Current	302 (34.2%)	276 (38.1%)	213 (40.5%)	
Smoking pack years	11.4 (0.4)	10.8 (0.4)	8.9 (0.5)	< 0.001
FEV1, %predicted	92.8 (0.4)	92.5 (0.6)	88.7 (0.7)	< 0.001
FVC, %predicted	93.3 (0.4)	95.0 (0.6)	90.3 (0.7)	< 0.001
FEV1/FVC, %	76.1 (0.2)	74.9 (0.3)	75.6 (0.4)	0.21
Airflow obstruction^c^	157 (20.7%)	167 (26.3%)	134 (31.0%)	0.02
Restrictive pattern^c^	125 (17.2%)	89 (15.9%)	93 (23.8%)	< 0.001
Self-reported chronic bronchitis	82 (9.2%)	78 (10.8%)	60 (11.4%)	0.59
Self-reported emphysema	33 (3.7%)	26 (3.6%)	19 (3.6%)	0.43
Self-reported asthma	77 (8.8%)	65 (9.0%)	44 (8.4%)	0.51
Medical record tuberculosis	133 (15.1%)	99 (13.7%)	60 (11.4%)	0.02

All analyses are weighted. Data are mean (SE) or *n* (% of tertile)

^a^Tertiles are range; calculated based on overall population; sum of inorganic and methylated species μg/g creatinine

^b^For continuous variables, ANOVA was used to calculate p-value; for categorical variables, chi-square test was used

^c^Fixed airflow obstruction: FEV1/FVC < 0.70

^c^Restrictive pattern: FEV1/FVC > 0.70 & FVC < 80% predicted

**Fig. 1:**
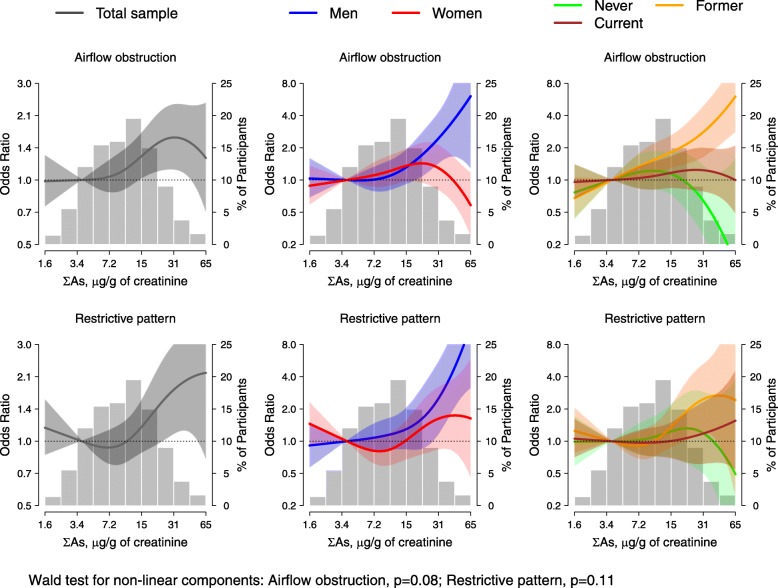
Dose-Response Relationship of Fixed Airflow Obstruction and Restrictive Pattern with Urinary Arsenic Concentrations. Solid lines and shaded areas surrounding the lines represent the weighted odds ratio and 95% confidence intervals of airflow obstruction (upper panels) and restrictive pattern (lower panels). Models were conducted in the total study sample (left panels), stratified by sex (middle panels), and stratified by smoking status (right panels). These models were adjusted for age, sex (except models stratified by sex), education, study site, smoking status (except models stratified by smoking status), smoking pack-year, eGFR, tuberculosis and BMI. Histograms in the background and right Y axis represent the distribution of urinary arsenic. The histograms were truncated by excluding 10 participants with urine arsenic concentrations above 65 μg/g of creatinine

After full adjustment (age, sex, education, site, smoking status, smoking pack-year, eGFR, tuberculosis, and BMI) (Table 3, model 3), the odds ratio [95% CI] comparing the highest to lowest arsenic tertile (≥14.0 vs. ≤7.0 μg/g creatinine) was 1.33 [0.99, 1.77] for obstruction and 1.34 [0.92, 1.96] for restrictive pattern. The corresponding OR [95%CI] for an interquartile range (IQR) increase of arsenic was 1.17 [0.99, 1.38] (*P* for trend 0.07) for obstruction and 1.27 [1.01, 1.60] (*P* for trend 0.04) for restrictive pattern (Table 3, model 3). Modelling urinary arsenic using flexible splines, showed positive and linear associations with restrictive pattern and airflow obstruction that were suggestive but nonsignificant in the complete sample (Fig. 1). Results were unchanged in analyses excluding 5 participants above the 99th percentile of %predicted FEV1 and FVC (results not shown). In a sensitivity analysis with further adjustment for diabetes, the OR for obstruction per change in arsenic IQR remained similar (1.17 [0.99, 1.40] (*P* for trend 0.07)), and for restrictive pattern the OR was attenuated (1.18 [0.93, 1.50] (*P* for trend 0.18)) (Table 3).

**Table 3:** Weighted Odds Ratio (95% Confidence Interval) of Airflow Obstruction and Restrictive Pattern, Defined Based on Fixed Ratios, by Urinary Arsenic Concentration

	Inorganic Plus Methylated Arsenic Species μg/g creatinine				*P*-trend^e^
	Tertile 1 ≤7.0^d^	Tertile 2 7.1–13.9^d^	Tertile 3 ≥14.0^d^	75th vs. 25th Percentile^f^	
Airflow obstruction^a^/Healthy^b^	157/600	167/469	134/298	458/1367	
Model 1	1.00 (Ref)	1.15 (0.93, 1.43)	1.45 (1.10, 1.91)	1.27 (1.08, 1.51)	0.005
Model 2	1.00 (Ref)	1.11 (0.89, 1.39)	1.34 (1.01, 1.77)	1.21 (1.01, 1.43)	0.03
Model 3	1.00 (Ref)	1.12 (0.90, 1.40)	1.33 (0.99, 1.77)	1.17 (0.99, 1.38)	0.07
Model 4	1.00 (Ref)	1.12 (0.90, 1.41)	1.33 (0.99, 1.79)	1.17 (0.99, 1.40)	0.07
Restrictive pattern^c^/ Healthy^b^	125/600	89/469	93/298	307/1367	
Model 1	1.00 (Ref)	0.92 (0.69, 1.23)	1.32 (0.92, 1.91)	1.25 (0.99, 1.57)	0.06
Model 2	1.00 (Ref)	0.91 (0.68, 1.22)	1.30 (0.90, 1.89)	1.23 (0.98, 1.55)	0.07
Model 3	1.00 (Ref)	0.92 (0.68, 1.23)	1.34 (0.92, 1.96)	1.27 (1.01, 1.60)	0.04
Model 4	1.00 (Ref)	0.88 (0.65, 1.19)	1.16 (0.78, 1.73)	1.18 (0.93, 1.50)	0.18

Model 1: adjusted for age, sex, education, site

Model 2: further adjusted for smoking status and smoking pack-year

Model 3: further adjusted for eGFR, tuberculosis, and BMI

Model 4: sensitivity analysis: further adjusted for diabetes

^a^Fixed airflow obstruction: FEV1/FVC < 0.70

^b^Healthy: FEV1/FVC > 0.70 & FVC > 80% predicted

^c^Restrictive pattern: FEV1/FVC > 0.70 & FVC < 80% predicted

^d^Tertiles are range; calculated based on overall population; sum of iAs, MMA, DMA μg/g creatinine

^e^P-trend calculated modeling log-arsenic as continuous

^f^Comparison of the 75th and 25th percentiles (interquartile range) of urinary arsenic concentrations (16.7 vs. 5.8 μg/g creatinine)

Using the LLN definition, obstruction was present in 7.1% (151/2132) and restrictive pattern in 6.9% (147/2132). The ORs for the association based on the LLN were stronger compared to the fixed ratio and were significant for obstruction (OR [95%CI] per IQR) (1.28 [1.02, 1.60] (*P* for trend 0.03) but non-significant for restriction (1.33 [0.90, 1.50] (*P* for trend 0.06) (Table 4).

**Table 4:** Weighted Odds Ratio (95% Confidence Interval) of Airflow Obstruction and Restrictive Pattern, Defined Based on the Lower Limit of Normal (LLN), by Urinary Arsenic Concentration (*N* = 2132)

	Inorganic Plus Methylated Arsenic Species μg/g creatinine				*P*-trend^d^
	Tertile 1 ≤7.0^e^	Tertile 2 7.1–13.9^e^	Tertile 3 ≥14.0^e^	75th vs. 25th Percentile^f^	
Airflow obstruction^a^/Healthy^c^	47/773	57/626	47/435	151/1834	
Model 1	1.00 (Ref)	1.15 (0.84, 1.58)	1.64 (1.16, 2.34)	1.47 (1.17, 1.88)	0.001
Model 2	1.00 (Ref)	1.11 (0.80, 1.53)	1.50 (1.05, 2.15)	1.38 (1.09, 1.76)	0.007
Model 3	1.00 (Ref)	1.08 (0.78, 1.50)	1.36 (0.94, 1.97)	1.28 (1.02, 1.60)	0.03
Model 4	1.00 (Ref)	1.07 (0.78, 1.49)	1.33 (0.92, 1.93)	1.26 (1.01, 1.59)	0.04
Restrictive pattern^b^/ Healthy^c^	62/773	42/626	43/435	147/1834	
Model 1	1.00 (Ref)	0.85 (0.58, 1.26)	1.45 (0.91, 2.30)	1.33 (1.00, 1.76)	0.05
Model 2	1.00 (Ref)	0.85 (0.58, 1.26)	1.41 (0.88, 2.25)	1.30 (0.98, 1.74)	0.07
Model 3	1.00 (Ref)	0.86 (0.58, 1.28)	1.42 (0.88, 2.28)	1.33 (0.90, 1.50)	0.06
Model 4	1.00 (Ref)	0.83 (0.56, 1.23)	1.23 (0.76, 2.00)	1.21 (0.90, 1.64)	0.22

Model 1: adjusted for age, sex, education, site

Model 2: further adjusted for smoking status and smoking pack-year

Model 3: further adjusted for eGFR, tuberculosis, and BMI

Model 4: sensitivity analysis: further adjusted for diabetes

^a^Airflow obstruction: FEV1/FVC < LLN

^b^Restrictive pattern: FEV1/FVC > LLN & FVC < LLN

^c^Healthy: FEV1/FVC > LLN and FVC > LLN

^d^*P*-trend calculated modeling log-arsenic as continuous

^e^Tertiles are range; calculated based on overall population; sum of iAs, MMA, DMA μg/g creatinine

^f^Comparison of the 75th and 25th percentiles (interquartile range) of urinary arsenic concentrations (16.7 vs. 5.8 μg/g creatinine)

**Fig. 2:**
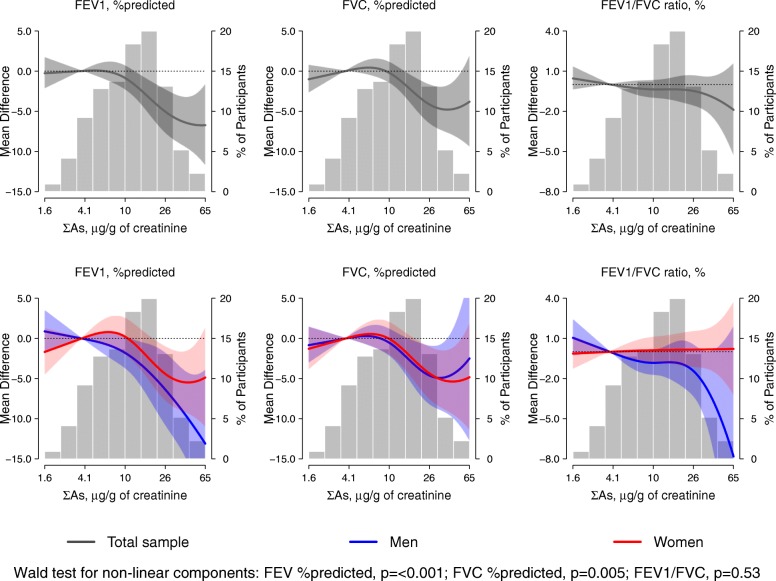
Dose-Response Relationship of Lung Function at Visit 2 (1993–1995) with Urinary Arsenic Concentrations. Solid lines and shaded areas surrounding the lines represent the weighted mean differences and 95% confidence intervals of FEV1% predicted (right panels), FVC % predicted (middle panels), and FEV1/FVC (right panels Models were conducted in the total study sample (upper panels) and stratified by sex (lower panels). These models were adjusted for age, sex (except models stratified by sex), education, study site, smoking status, smoking pack-year, eGFR, tuberculosis and BMI. Histograms in the background and right Y axis represent the distribution of urinary arsenic. The histograms were truncated by excluding 10 participants with urine arsenic concentrations above 65 μg/g of creatinine

The mean difference [95% CI] for FEV1%predicted for an IQR change in urinary arsenic was − 1.39 [− 2.51, − 0.25] (*P* for trend 0.02), although the trend was non-linear by tertile (Table 5) and flexible splines (Fig. 2). The %predicted association remained significant after further adjustment for diabetes in the sensitivity analyses (Additional file 1: Table S1). For FVC %predicted, the mean difference [95% CI] per IQR change in arsenic was − 1.13 [− 2.21, − 0.05] (*P* for trend 0.04). Among the healthy group, the mean difference for FEV1%predicted and FVC %predicted both became non-significant (Table 5) and remained non-significant in the sensitivity analysis (Additional file 1: Table S1). Wald test results for non-linear components of the spline model were *p* = < 0.001 for FEV1%predicted and *p* = 0.005 for FVC %predicted. No association was found between arsenic and FEV1/FVC. Using crude FEV1 and FVC measures (mL) the mean differences were significant (Table 5).

**Table 5:** Weighted Mean Difference (95% Confidence Interval) of Lung Function at Visit 2 (1993–1995) by Urinary Arsenic Concentration at Baseline (1989–1991)

	*N*	Inorganic Plus Methylated Arsenic Species μg/g creatinine				*P*-trend^d^
		Tertile 1 ≤7.0^b^	Tertile 2 7.1–13.9^b^	Tertile 3 ≥14.0^b^	75th vs. 25th Percentile^c^	
FEV1, % predicted						
All	2132	0 (Ref)	0.92 (−0.52, 2.37)	−1.64 (−3.60, 0.32)	−1.39 (−2.51, −0.25)	0.02
Healthy^a^	1367	0 (Ref)	0.67 (−0.86, 2.19)	−0.49 (−2.58, 1.61)	0.85 (0.27, 2.74)	0.80
FVC, % predicted						
All	2132	0 (Ref)	2.09 (0.72, 3.47)	−1.01 (−2.85, 0.83)	−1.13 (−2.21, − 0.05)	0.04
Healthy^a^	1367	0 (Ref)	1.15 (− 0.23, 2.53)	− 0.73 (− 2.60, 1.14)	0.70 (0.24, 2.02)	0.50
FEV1/FVC (%)						
All	2132	0 (Ref)	−0.62 (−1.26, 0.002)	− 0.16 (− 1.01, 0.69)	0.09 (− 0.46, 0.66)	0.74
Healthy^a^	1367	0 (Ref)	− 0.31 (− 0.85, 0.25)	0.26 (− 0.49, 1.01)	1.21 (0.76, 1.94)	0.42
FEV1, mL						
All	2132	0 (Ref)	0.007 (−0.04, 0.06)	−0.09 (− 0.15, − 0.03)	−0.07 (− 0.11, − 0.03)	< 0.001
Healthy^a^	1367	0 (Ref)	0.003 (− 0.05, 0.06)	−0.06 (− 0.14, 0.01)	−0.03 (− 0.07, − 0.003)	0.07
FVC, mL						
All	2132	0 (Ref)	0.06 (−0.004, 0.11)	−0.10 (− 0.17, − 0.02)	−0.07 (− 0.12, − 0.03)	0.001
Healthy^a^	1367	0 (Ref)	0.02 (− 0.05, 0.09)	−0.09 (− 0.19, − 0.0001)	−0.05 (− 0.10, − 0.004)	0.03

Adjusted for age, sex, education, site, smoking status, smoking pack-year, eGFR, tuberculosis, and BMI

^a^Healthy: FEV1/FVC > 0.70 & FVC > 80% predicted

^b^Tertiles are range; calculated based on overall population; sum of inorganic and methylated species μg/g creatinine

^c^Comparison of the 75th and 25th percentiles (interquartile range) of the sum inorganic and methylated urinary arsenic concentrations (16.7 vs. 5.8 μg/g creatinine)

^d^*P*-trend calculated modeling log-arsenic as continuous

We found no effect modification for the association of arsenic with obstruction/restrictive pattern by age, BMI, or diabetes (Additional file 1: Table S2). By sex, effect modification was significant for obstruction (*P* = 0.003), with an association found in men (OR [95%CI]) (1.47 [1.07, 2.06]) but not significant in women (1.07 [0.82, 1.33]). By smoking status, the association with arsenic was strongest in former smokers both for obstruction (1.74 [1.20, 2.55]) and restrictive pattern (1.34 [0.82, 2.17]) compared to never or current smokers, but confidence intervals overlapped in both analyses. Urinary relative proportions of iAs, MMA, and DMA were not associated with obstruction/restrictive pattern (Additional file 1: Table S4).

Urinary arsenic was inversely associated with cough (OR [95%CI] per IQR) (0.78 [0.65, 0.93]), but not with frequent cough (4–6x/day) or production of phlegm (Table 6). There was no association between arsenic and shortness of breath, but arsenic was positively associated with stopping for breath while walking (1.41 [1.19, 1.69]) (Table 6). Urinary arsenic was positively associated with emphysema (OR [95%CI] per IQR) (1.66 [1.29, 2.15]); inversely associated with asthma (0.76 [0.61, 0.96]) and not associated with chronic bronchitis (Additional file 1: Table S3).

**Table 6:** Weighted Odds Ratio (95% Confidence Interval) of Respiratory Symptom by Urinary Arsenic Concentration (*N* = 2132)

	Inorganic Plus Methylated Arsenic Species μg/g creatinine				*P*-trend^b^
	Tertile 1 ≤7.0^a^	Tertile 2 7.1–13.9^a^	Tertile 3 ≥14.0^a^	75th vs. 25th Percentile	
Cough^c^/No cough	191/690	169/555	116/406	476/1651	
Model 1	1.00 (Ref)	0.87 (0.70, 1.09)	0.69 (0.51, 0.93)	0.82 (0.69, 0.98)	0.03
Model 2	1.00 (Ref)	0.86 (0.69, 1.06)	0.64 (0.48, 0.87)	0.79 (0.66, 0.93)	0.006
Model 3	1.00 (Ref)	0.84 (0.68, 1.05)	0.63 (0.47, 0.86)	0.78 (0.65, 0.93)	0.005
Model 4	1.00 (Ref)	0.84 (0.68, 1.05)	0.63 (0.46, 0.85)	0.77 (0.65, 0.92)	0.004
Cough 4–6x per day^d^/No	114/768	111/614	79/446	304/1828	
Model 1	1.00 (Ref)	0.97 (0.73, 1.28)	0.86 (0.59, 1.26)	0.96 (0.78, 1.18)	0.71
Model 2	1.00 (Ref)	0.94 (0.71, 1.24)	0.79 (0.54, 1.16)	0.91 (0.73, 1.12)	0.36
Model 3	1.00 (Ref)	0.94 (0.71, 1.25)	0.80 (0.54, 1.17)	0.92 (0.74, 1.13)	0.44
Model 4	1.00 (Ref)	0.94 (0.71, 1.25)	0.81 (0.55, 1.18)	0.93 (0.75, 1.15)	0.48
Phlegm^e^/No	117/765	114/611	70/455	301/1831	
Model 1	1.00 (Ref)	1.19 (0.95, 1.49)	1.06 (0.77, 1.47)	1.09 (0.90, 1.31)	0.37
Model 2	1.00 (Ref)	1.16 (0.93, 1.46)	0.99 (0.71, 1.37)	1.03 (0.86, 1.25)	0.71
Model 3	1.00 (Ref)	1.18 (0.94, 1.49)	1.01 (0.73, 1.41)	1.05 (0.87, 1.27)	0.57
Model 4	1.00 (Ref)	1.18 (0.94, 1.48)	1.01 (0.72, 1.40)	1.05 (0.87, 1.27)	0.59
Shortness of breath^f^/No	369/503	363/355	243/275	975/1133	
Model 1	1.00 (Ref)	1.16 (0.98, 1.37)	0.90 (0.72, 1.13)	1.02 (0.88, 1.17)	0.81
Model 2	1.00 (Ref)	1.16 (0.98, 1.37)	0.88 (0.70, 1.11)	1.00 (0.87, 1.15)	0.97
Model 3	1.00 (Ref)	1.17 (0.98, 1.39)	0.93 (0.73, 1.18)	1.08 (0.93, 1.23)	0.34
Model 4	1.00 (Ref)	1.18 (0.99, 1.40)	0.94 (0.74, 1.20)	1.08 (0.94, 1.25)	0.28
Stop for breath^g^/No	101/781	151/574	103/422	355/1777	
Model 1	1.00 (Ref)	1.67 (1.35, 2.07)	1.56 (1.18, 2.06)	1.33 (1.11, 1.59)	0.002
Model 2	1.00 (Ref)	1.66 (1.34, 2.05)	1.52 (1.14, 2.01)	1.30 (1.08, 1.55)	0.005
Model 3	1.00 (Ref)	1.76 (1.42, 2.19)	1.68 (1.26, 2.24)	1.41 (1.19, 1.69)	< 0.001
Model 4	1.00 (Ref)	1.76 (1.41, 2.19)	1.64 (1.23, 2.20)	1.40 (1.17, 1.67)	< 0.001

Model 1: adjusted for age, sex, education, site

Model 2: further adjusted for smoking status and smoking pack-year

Model 3: further adjusted for eGFR, tuberculosis, and BMI

Model 4: sensitivity analysis: further adjusted for diabetes

^a^Tertiles are range; calculated based on overall population; sum of inorganic and methylated species μg/g creatinine

^b^*P*-trend calculated modeling log-arsenic as continuous

^c^Do you usually have a cough?

^d^Do you usually cough as much as 4–6 times/day, 4 or more days/week?

^e^Do you usually bring up phlegm when you cough?

^f^Are you troubled by shortness of breath when hurrying on the level or walking up a slight hill?

^g^Do you ever have to stop for breath while walking about 100 yards or a few minutes on the level?

## Discussion

Exposure to low-moderate levels of inorganic arsenic was associated with increased odds of fixed ratio restrictive lung pattern, lower FEV1 and lower FVC, borderline associated with fixed ratio obstruction, and not associated with FEV1/FVC. The associations based on the LLN became stronger and significant for obstruction and stronger but non-significant for restrictive pattern. Arsenic was also associated with stopping for breath while walking and with higher self-reported emphysema. The association with restrictive pattern is consistent with recent meta-analysis findings that suggested low-level arsenic exposure is a restrictive lung disease risk factor [4]. There is debate over using the fixed ratio definition of obstruction, which can potentially lead to over-diagnoses in older individuals [18, 19]. However, there are also limitations with LLN-defined obstruction, which can underestimate airflow obstruction [20]. The stronger but non-significant effect estimates we see for the association between arsenic and LLN-defined restrictive pattern may be due to a more specific definition and exclusion of less severe cases.

Restrictive pattern findings remained significant after adjustment for smoking (status and pack-years), a major risk factor for reduced pulmonary function [21, 22]. In a sensitivity analysis (results not shown), we adjusted for additional adiposity factors (% body fat, waist circumference) to account for mechanical constraints of obesity-related lung restriction [23] with consistent findings. Adjustment for diabetes, however, attenuated the association, which became non-significant. The definitive direction as well as the exact pathophysiological mechanism to explain the association between diabetes and lung function is not known [24]; in the Strong Heart Study, impaired lung function presented before the development of diabetes [25]. Previous similar studies have not adjusted for diabetes, but there is a large body of evidence suggesting that chronic arsenic exposure can contribute to diabetes development [26], and diabetes could be in the causal pathway between arsenic and restrictive lung pattern. Lung restriction in diabetes can result from chronic low-grade inflammation of the lung tissue; lung volume has been found to inversely correlate with the level of systemic inflammation, [24] with a restrictive pattern of lung function loss associated with systemic inflammation [27].

There is consistent evidence that increasing arsenic exposure is associated with reports of coughing and breathing problems [5]. However, we only found a positive association between arsenic and with the need to stop for breath and a reduced odds of cough. One study in the US also found lower odds of chronic cough in participants with greater than the 80th (< 17.23 μg/L) arsenic percentile compared to those with less than the 20th (< 3.52 μg/L) percentile [6]. The same study reported greater odds of self-reported emphysema, similar to our findings, among those with the highest quartile of urinary arsenic compared to the lowest, but results were non-significant [6]. Four studies have examined arsenic and chronic bronchitis; three found a greater odds [28–30] and one found reduced odds [6].

Despite epidemiologic evidence, little is known regarding arsenic-induced effects on airway physiology [31, 32]. Rather than a direct toxic effect of arsenic on the lung, an inflammation-mediated immunologic basis is suggested [33], as arsenic is known to alter key functions of the innate and adaptive immune system [34–37]. One possible mechanism is aberrant airway remodeling targeted by arsenic following activation of inflammatory mediators. Airway remodeling has been linked to the equilibrium between proteases matrix metalloproteinase-9 (MMP-9) and its inhibitors, receptor for advanced glycation end products (RAGE) [38]. Loss of the soluble form of RAGE, sRAGE, is related to functional changes of pulmonary cell types, with consequences of fibrotic disease. Arsenic may change RAGE gene expression by altering the promoter region methylation or by affecting transcriptional regulators of RAGE. In humans, sputum sRAGE levels were negatively correlated with urinary arsenic levels, similar to animal models [39]. In vitro models have shown arsenic exposure increases activity and expression of MMP-9 in airway epithelial cells [40].

This study had several limitations. We measured urinary arsenic levels in a single sample at baseline, while spirometric measurements were taken at Visit 2. However, the temporal stability of arsenic levels in drinking water and urine has been shown in this population [11]. Spirometry was originally performed for better prediction of cardiovascular disease [13]. We did not have total lung capacity measurements, often not available for large population screenings, and could not confirm restriction presence. We also could not confirm the presence of obstructive disease without post-bronchodilator spirometry. Thus, we cannot discard the possibility that the association we found may be due to mixed ventilatory defect. Outcome misclassification could have occurred from inaccurate recall of disease diagnosis. The reason we saw a significant relationship between arsenic and obstruction only in former smokers is unknown. A few studies have reported similar findings, with authors suggesting the toxic effects of smoking could be masking those of arsenic [28, 41]. A recent meta-analysis found the association between arsenic and FVC to be slightly stronger among non-smokers than smokers, also for reasons unknown [4]. This finding, too, is surprising, as generally the quickest benefit after quitting cigarette smoking is improvement in lung function. This further points to the possibility that active smoking’s toxic effects could be masking those of arsenic; however, this is speculative.

Strengths of this study include having American Indian reference values derived from the SHS cohort [12]. This is important as anthropomorphic differences vary between ethnic groups, and NHANES III, from which normative values are generated, did not include American Indians. The reference values allowed for results to be evaluated for abnormalities against predicted values for better interpretation of results. Other major strengths include the community-based sample, standardized spirometry, and extensive data on potential confounders.

## Conclusions

Our study provides evidence of an association between low-moderate arsenic exposure and a spirometric restrictive pattern, airflow obstruction (especially based on the LLN), and higher self-reported emphysema and stopping for breath. No other study has evaluated the association between arsenic exposure and individual spirometric lung function in American Indians, US population, or population exposed to low-moderate arsenic levels. Research in additional populations is needed to confirm the association, including evaluation of relevant subclinical and pathophysiological outcomes. This could include repeated urinary arsenic measurement and diagnostic testing, like computed tomography scan, to better assess patterns of lung disease.

## Supplementary information

**Additional file 1: Table S1.** Sensitivity Analysis: adjustment for diabetes. Weighted Mean Difference (95% Confidence Interval) of Lung Function at Visit 2 (1993-1995) by Urinary Arsenic Concentration* at Baseline (1989-1991). **Table S2.** Weighted Odds Ratios (95% Confidence Interval) for Airflow Obstruction and Restrictive Pattern, Defined Based on Fixed Ratios, when an Interquartile Range* of Urinary Arsenic Concentration is Compared, by Participant Characteristics at Baseline. **Table S3.** Weighted Odds Ratio (95% Confidence Interval) of Self-reported Emphysema, Chronic Bronchitis, or Asthma by Urine Arsenic Tertile Concentration. **Table S4.** Weighted Odds Ratio (95% Confidence Interval) of Airflow Obstruction and Restrictive Pattern, Defined Based on Fixed Ratios, by 5% Change in Urinary Arsenic Metabolites*.

## Data Availability

Strong Heart Study data are shared with researchers following Resource and Data Sharing Policies, which include review and approval from the Strong Heart Study Steering Committee and Strong Heart Study participating tribes.

## Abbreviations

BMI: Body mass index
DMA: Dimethylarsinate
FEV1: Forced expiratory volume in one second
FVC: Reduced forced vital capacity
iAs: Inorganic arsenic
IQR: Interquartile range
LLN: Lower limit of normal, the 5th percentile of the frequency distribution of reference values
MMA: Methylarsonate
OR: Odds ratio
SHS: Strong Heart Study
