# Correction to: Low-moderate arsenic exposure and respiratory health in American Indian communities in the Strong Heart Study

**DOI:** 10.1186/s12940-020-00576-z

**Published:** 2020-02-26

**Authors:** Martha Powers, Tiffany R. Sanchez, Maria Grau-Perez, Fawn Yeh, Kevin A. Francesconi, Walter Goessler, Christine M. George, Christopher Heaney, Lyle G. Best, Jason G. Umans, Robert H. Brown, Ana Navas-Acien

**Affiliations:** 1 Department of Environmental Health and Engineering, Johns Hopkins Bloomberg School of Public Health, 615 N. Wolfe St, Baltimore, MD USA; 2 Department of Environmental Health Sciences, Columbia University Mailman School of Public Health, New York, NY USA; 3 Center for American Indian Health Research, University of Oklahoma Health Sciences Center, College of Public Health, Oklahoma City, OK USA; 4 Institute of Chemistry - Analytical Chemistry, Graz, Austria; 5 Department of International Health, Johns Hopkins Bloomberg School of Public Health, Baltimore, MD USA; 6 Department of Epidemiology, Johns Hopkins Bloomberg School of Public Health, Baltimore, MD USA; 7 Missouri Breaks Industries Research, Inc, Eagle Butte, SD USA; 8 MedStar Health Research Institute, Hyattsville, MD, USA, Georgetown-Howard Universities Center for Clinical and Translational Science, Washington, DC USA; 9 Department of Anesthesiology and Critical Care Medicine, Johns Hopkins School of Medicine, Baltimore, MD USA

**Correction to: Environ Health**

**https://doi.org/10.1186/s12940-019-0539-6**

The original version of this article [1], published on 28 November 2019, contained incorrect title. In this Correction the affected part of the article is shown.

Incorrect title:

Low-moderate arsenic exposure and respiratory in American Indian communities in the Strong Heart St

Correct title:

Low-moderate arsenic exposure and respiratory health in American Indian communities in the Strong Heart Study

In this correction article the title is shown correct.

## References

[1] Powers, et al. Low-moderate arsenic exposure and respiratory health in American Indian communities in the Strong Heart Study. Environ Health. 2019;18:104. 10.1186/s12940-019-0539-6. 10.1186/s12940-019-0539-6 PMC6883619 31779614

