# Supplementary material for: Low-moderate arsenic exposure and respiratory in American Indian communities in the Strong Heart Study
Source: Environ Health. 2019 Nov 28;18:104. doi: 10.1186/s12940-019-0539-6 (PMC6883619; doi:10.1186/s12940-019-0539-6)
Supplement: Supplementary file 1 — Additional file 1: Table S1. Sensitivity Analysis: adjustment for diabetes. Weighted Mean Difference (95% Confidence Interval) of Lung Function at Visit 2 (1993-1995) by Urinary Arsenic Concentration* at Baseline (1989-1991). Table S2. Weighted Odds Ratios (95% Confidence Interval) for Airflow Obstruction and Restrictive Pattern, Defined Based on Fixed Ratios, when an Interquartile Range* of Urinary Arsenic Concentration is Compared, by Participant Characteristics at Baseline. Table S3. Weighted Odds Ratio (95% Confidence Interval) of Self-reported Emphysema, Chronic Bronchitis, or Asthma by Urine Arsenic Tertile Concentration. Table S4. Weighted Odds Ratio (95% Confidence Interval) of Airflow Obstruction and Restrictive Pattern, Defined Based on Fixed Ratios, by 5% Change in Urinary Arsenic Metabolites*. [file 12940_2019_539_MOESM1_ESM.docx]

**Additional file Tables.**

**Table S1. Sensitivity Analysis: adjustment for diabetes**. Weighted Mean Difference (95% Confidence Interval) of Lung Function at Visit 2 (1993-1995) by Urinary Arsenic Concentration* at Baseline (1989-1991)

|  | **N** | **Inorganic Plus Methylated Arsenic Species**  **µg/g creatinine** | | | | **P-trend**** |
| --- | --- | --- | --- | --- | --- | --- |
|  |  | **Tertile 1**  ≤7.0* | **Tertile 2**  7.1-13.9* | **Tertile 3**  ≥14.0* | **75^th^ vs. 25^th^ Percentile**^‡^ |  |
| **FEV1, % predicted** |  |  |  |  |  |  |
| All | 2132 | 0 (Ref) | 0.91 (-0.56, 2.39) | -1.44 (-3.44, 0.56) | -1.28 (-2.39, -0.17) | 0.02 |
| Healthy **^α^** | 1367 | 0 (Ref) | 0.67 (-0.86, 2.20) | -0.47 (-2.60, 2.20) | 0.87 (0.27, 2.80) | 0.82 |
|  |  |  |  |  |  |  |
| **FVC, % predicted** |  |  |  |  |  |  |
| All | 2132 | 0 (Ref) | 2.16 (0.77, 3.56) | -0.54 (-2.41, 1.34) | -0.85 (-1.91, 0.22) | 0.12 |
| Healthy **^α^** | 1367 | 0 (Ref) | 1.20 (-0.20, 2.59) | -0.49 (-2.40, 1.43) | 0.79 (0.27, 2.32) | 0.67 |
|  |  |  |  |  |  |  |
| **FEV1/FVC (%)** |  |  |  |  |  |  |
| All | 2132 | 0 (Ref) | -0.70 (-1.36, -0.05) | -0.36 (-1.23, 0.52) | -0.01 (-0.58, 0.55) | 0.97 |
| Healthy **^α^** | 1367 | 0 (Ref) | -0.35 (-0.89, 0.20) | 0.08 (-0.66, 0.81) | 1.10 (0.69, 1.74) | 0.68 |

**^α^** Healthy: FEV1/FVC >0.70 & FVC >80% predicted

Adjusted for age, sex, education, site, smoking status, smoking pack-year, eGFR, tuberculosis, BMI, and diabetes

*Tertiles are range; calculated based on overall population; sum of inorganic and methylated species µg/g creatinine

**^‡^** Comparison of the 75^th^ and 25^th^ percentiles (interquartile range) of the sum inorganic and methylated urinary arsenic concentrations (16.7 vs. 5.8 µg/g creatinine)

**P-trend calculated modeling log-arsenic as continuous

**Table S2.** Weighted Odds Ratios (95% Confidence Interval) for Airflow Obstruction and Restrictive Pattern, Defined Based on Fixed Ratios, when an Interquartile Range* of Urinary Arsenic Concentration is Compared, by Participant Characteristics at Baseline

|  | Airflow obstruction **^α^** | | | | Restrictive pattern **^α^** | | | |
| --- | --- | --- | --- | --- | --- | --- | --- | --- |
|  | FEV1/FVC <0.70 | | | | FEV1/FVC >0.70 & FVC <80% predicted | | | |
| **Variable** | N | Odds Ratio | 95% CI | P for interaction** | N | Odds Ratio | 95% CI | P for interaction** |
| Sex |  |  |  | 0.003 |  |  |  | 0.82 |
| Male | 739 | 1.47 | (1.07, 2.06) |  | 608 | 1.10 | (0.69, 1.76) |  |
| Female | 1086 | 1.07 | (0.82, 1.33) |  | 1066 | 1.23 | (0.90, 1.69) |  |
|  |  |  |  |  |  |  |  |  |
| Age^‡^, years |  |  |  | 0.52 |  |  |  | 0.43 |
| <55.7 | 977 | 1.31 | (1.01, 1.71) |  | 965 | 1.30 | (0.91, 1.86) |  |
| ≥55.7 | 848 | 1.12 | (0.85, 1.47) |  | 709 | 1.06 | (0.74, 1.54) |  |
|  |  |  |  |  |  |  |  |  |
| Smoking status |  |  |  | 0.12 |  |  |  | 0.03 |
| Never | 564 | 0.88 | (0.57, 1.37) |  | 560 | 0.99 | (0.60, 1.64) |  |
| Former | 577 | 1.74 | (1.20, 2.55) |  | 534 | 1.34 | (0.82, 2.17) |  |
| Current | 684 | 1.10 | (0.81, 1.51) |  | 580 | 1.16 | (0.79, 1.73) |  |
|  |  |  |  |  |  |  |  |  |
| BMI, kg/m^2^ |  |  |  | 0.15 |  |  |  | 0.31 |
| <25 | 317 | 1.51 | (0.99, 2.29) |  | 220 | 0.91 | (0.39, 2.11) |  |
| ≥25 - <30 | 646 | 1.29 | (0.93, 1.78) |  | 578 | 1.67 | (1.09, 2.57) |  |
| ≥30 | 862 | 0.89 | (0.62, 1.29) |  | 876 | 1.04 | (0.72, 1.51) |  |
|  |  |  |  |  |  |  |  |  |
| Diabetes |  |  |  | 0.84 |  |  |  | 0.60 |
| Yes | 707 | 1.05 | (0.71, 1.57) |  | 724 | 1.17 | (0.82, 1.69) |  |
| No | 1,118 | 1.23 | (0.99, 1.54) |  | 950 | 1.08 | (0.74, 1.59) |  |

*****Interquartile range of the sum inorganic and methylated urinary arsenic concentrations was 5.8 to 16.7 µg/g creatinine

**^α^** Compared to Healthy: FEV1/FVC >0.70 & FVC >80% predicted

******ORs were stratified by each subgroup of interest, and associated *P* values for interaction were obtained from models with interaction terms and using Wald tests for multiple coefficients

Models were adjusted for sex, age, education, site, smoking status, cigarette pack-years, body mass index, estimated glomerular filtration rate, and tuberculosis.

^‡^ Mean age.

**Table S3**. Weighted Odds Ratio (95% Confidence Interval) of Self-reported Emphysema, Chronic Bronchitis, or Asthma by Urine Arsenic Tertile Concentration

|  | **Inorganic Plus Methylated Arsenic Species µg/g creatinine** | | | **75^th^ vs. 25^th^ Percentile**^‡^ | **P-trend**** |
| --- | --- | --- | --- | --- | --- |
|  | **Tertile 1**  ≤7.0* | **Tertile 2**  7.1-13.9* | **Tertile 3**  ≥14.0* |  |  |
| **Chronic Bronchitis /None** (N=2,116) | 82/797 | 78/638 | 60/461 | 220/1896 |  |
| Model 1 | 1.00 (Ref) | 1.15 (0.85, 1.56) | 1.19 (0.79, 1.81) | 1.13 (0.90, 1.44) | 0.29 |
| Model 2 | 1.00 (Ref) | 1.14 (0.84, 1.54) | 1.16 (0.76, 1.75) | 1.11 (0.88, 1.40) | 0.37 |
| Model 3 | 1.00 (Ref) | 1.14 (0.84, 1.56) | 1.20 (0.79, 1.83) | 1.16 (0.92, 1.47) | 0.22 |
| Model 4 | 1.00 (Ref) | 1.14 (0.84, 1.55) | 1.17 (0.76, 1.79) | 1.15 (0.90, 1.46) | 0.27 |
|  |  |  |  |  |  |
| **Emphysema/None**  (N=2,116) | 33/847 | 26/692 | 19/499 | 78/2038 |  |
| Model 1 | 1.00 (Ref) | 1.23 (0.88, 1.74) | 2.04 (1.35, 3.08) | 1.76 (1.38, 2.27) | <0.001 |
| Model 2 | 1.00 (Ref) | 1.18 (0.83, 1.67) | 1.79 (1.16, 2.77) | 1.64 (1.27, 2.09) | <0.001 |
| Model 3 | 1.00 (Ref) | 1.18 (0.83, 1.67) | 1.83 (1.18, 2.83) | 1.66 (1.29, 2.15) | <0.001 |
| Model 4 | 1.00 (Ref) | 1.17 (0.82, 1.66) | 1.79 (1.16, 2.78) | 1.66 (1.27, 2.13) | <0.001 |
|  |  |  |  |  |  |
| **Asthma/None**  (N=2,117) | 77/800 | 65/653 | 44/478 | 186/1931 |  |
| Model 1 | 1.00 (Ref) | 0.91 (0.67, 1.25) | 0.61 (0.40, 0.94) | 0.76 (0.60, 0.96) | 0.02 |
| Model 2 | 1.00 (Ref) | 0.90 (0.66, 1.23) | 0.60 (0.39, 0.93) | 0.75 (0.60, 0.95) | 0.02 |
| Model 3 | 1.00 (Ref) | 0.90 (0.66, 1.23) | 0.61 (0.40, 0.95) | 0.76 (0.60, 0.96) | 0.02 |
| Model 4 | 1.00 (Ref) | 0.90 (0.66, 1.24) | 0.63 (0.41, 0.97) | 0.77 (0.61, 0.98) | 0.03 |

*Tertiles are range; calculated based on overall population; sum of inorganic and methylated species µg/g creatinine

**P-trend calculated modeling log-arsenic as continuous

Model 1: adjusted for age, sex, education, site

Model 2: further adjusted for smoking status and smoking pack-year

Model 3: further adjusted for eGFR, tuberculosis, and BMI

Model 4: sensitivity analysis: further adjusted for diabetes

**Table S4**. Weighted Odds Ratio (95% Confidence Interval) of Airflow Obstruction and Restrictive Pattern, Defined Based on Fixed Ratios, by 5% Change in Urinary Arsenic Metabolites*

|  | %iAs | %MMA | %DMA |
| --- | --- | --- | --- |
| **Airflow obstruction/Healthy**  (439/1,282) |  |  |  |
| Model 3 | 1.04 (0.93, 1.15) | 0.92 (0.83, 1.02) | 1.02 (0.96, 1.09) |
| Model 4 | 1.04 (0.93, 1.15) | 0.92 (0.83, 1.02) | 1.04 (0.93, 1.15) |
|  |  |  |  |
| **Restrictive pattern/Healthy**  (280/1,282) |  |  |  |
| Model 3 | 0.95 (0.81, 1.12) | 0.88 (0.77, 1.00) | 1.07 (0.98, 1.17) |
| Model 4 | 0.94 (0.80, 1.11) | 0.93 (0.81, 1.07) | 1.05 (0.96, 1.14) |

*sum of inorganic and methylated species µg/g creatinine

Airflow obstruction: FEV1/FVC <0.70

Healthy: FEV1/FVC >0.70 & FVC >80% predicted

Restrictive pattern: FEV1/FVC >0.70 & FVC <80% predicted

Model 3: adjusted for age, sex, education, site, smoking status, smoking pack-year, eGFR, tuberculosis, and BMI

Model 4: sensitivity analysis: further adjusted for diabetes
